# Bioluminescent flashes drive nighttime schooling behavior and synchronized swimming dynamics in flashlight fish

**DOI:** 10.1371/journal.pone.0219852

**Published:** 2019-08-14

**Authors:** David F. Gruber, Brennan T. Phillips, Rory O’Brien, Vivek Boominathan, Ashok Veeraraghavan, Ganesh Vasan, Peter O’Brien, Vincent A. Pieribone, John S. Sparks

**Affiliations:** 1 Department of Natural Sciences, City University of New York, Baruch College, New York, New York, United States of America; 2 PhD Program in Biology, The Graduate Center, City University of New York, New York, New York, United States of America; 3 Sackler Institute for Comparative Genomics, American Museum of Natural History, New York, New York, United States of America; 4 Department of Ocean Engineering, University of Rhode Island, Narragansett, Rhode Island, United States of America; 5 Department of Cellular and Molecular Physiology, The John B. Pierce Laboratory, Yale University School of Medicine, New Haven, Connecticut, United States of America; 6 Rice University, Department of Electrical and Computer Engineering, Houston, Texas, United States of America; 7 Department of Ichthyology, Division of Vertebrate Zoology, American Museum of Natural History, New York, New York, United States of America; University of Auckland, NEW ZEALAND

## Abstract

Schooling fishes, like flocking birds and swarming insects, display remarkable behavioral coordination. While over 25% of fish species exhibit schooling behavior, nighttime schooling has rarely been observed or reported. This is due to vision being the primary modality for schooling, which is corroborated by the fact that most fish schools disperse at critically low light levels. Here we report on a large aggregation of the bioluminescent flashlight fish *Anomalops katoptron* that exhibited nighttime schooling behavior during multiple moon phases, including the new moon. Data were recorded with a suite of low-light imaging devices, including a high-speed, high-resolution scientific complementary metal-oxide-semiconductor (sCMOS) camera. Image analysis revealed nighttime schooling using synchronized bioluminescent flashing displays, and demonstrated that school motion synchrony exhibits correlation with relative swim speed. A computer model of flashlight fish schooling behavior shows that only a small percentage of individuals need to exhibit bioluminescence in order for school cohesion to be maintained. Flashlight fish schooling is unique among fishes, in that bioluminescence enables schooling in conditions of no ambient light. In addition, some members can still partake in the school while not actively exhibiting their bioluminescence. Image analysis of our field data and model demonstrate that if a small percentage of fish become motivated to change direction, the rest of the school follows. The use of bioluminescence by flashlight fish to enable schooling in shallow water adds an additional ecological application to bioluminescence and suggests that schooling behavior in mesopelagic bioluminescent fishes may be also mediated by luminescent displays.

## Introduction

It is estimated that over a quarter of the world’s fish species school throughout their lives [1] and many schooling fishes spend a large portion of their lives in schools. Behavioral and evolutionary studies of schooling fishes indicate that group membership is advantageous, conferring a lower risk of predation [2–5], greater access to food resources [6], better mate choice [7] and reduced cost of transport [8]. Parr first proposed that schooling fish have attraction and repulsion forces that maintain the distance between neighboring individuals [9]. In this study, we utilize the definition of “schooling” as the tendency of individuals to synchronize their behavior and swim in an oriented, polarized manner relative to one another, whereas “shoaling” is herein defined to be a loosely organized group of fish [10,11].

Schooling fishes rely on their ability to sense one another. Vision is widely accepted as the most paramount schooling modality [9,12–14]; with fish schools dissipating below critical illumination levels [15,16]. These minimal light levels have been defined for several species, such as *Brevoortia patronus* (gulf menhaden silverside), *Engraulis mordax* (California anchovy), and *Trachurus symmetricus* (jack mackerel), via aquarium studies [17–19], and there is correlation between threshold light intensity for schooling and eye diameter for a number of different fish species [17]. Light intensity thresholds for schooling have also been shown to vary depending on a species’ lifecycle [20,21]. At twilight, fish schools gradually lose their shape and the distance between individuals rises until, at night, some species form amorphous loose aggregations consisting of what formerly comprised many different schools [4]. The ability to sense hydrodynamic forces through the lateral line also plays a role in schooling behavior [22–26], as fish tend to take up positions that allow them to remain close to their neighbors without experiencing excessive turbulence.

**Fig 1:**
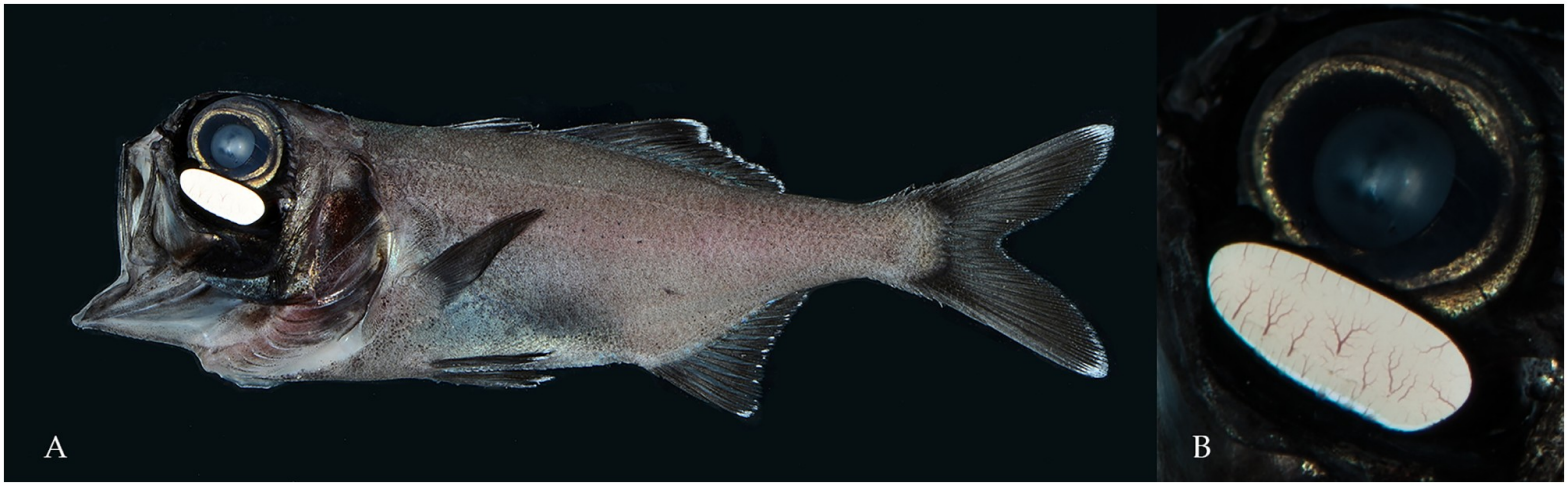
Representative *Anomalops katoptron* imaged in this study. A) Image of adult *Anomalops katoptron*; B) macro of the bioluminescent organ depicting highly vascularized structure necessary for providing oxygen to bioluminescent symbiotic bacteria.

Observations of flashlight fish (Anomalopidae) in their natural environment at night has captured the attention of scientists for centuries and was eloquently described by Dr. Eugenie Clark as, “like floating among the stars.” [27] Bioluminescence in flashlight fishes is driven via symbiotic bioluminescent bacteria grown in specialized tubes within the fish’s subocular bioluminescent organs that assist in enhancing light output [28,29] (Fig 1). The bioluminescent bacteria are contained within a mass of parallel tubules up to 1 mm in length and 30–40 microns in diameter. The tubules are aligned at right angles to the surface of the organ and the base of each tubule abuts a reflector, which in *Anomalops* is comprised of two parts [30]. The main interior reflector covers most of the inner surface of the organ and is composed of stacks of guanine crystals that lie parallel to the surface. As the bioluminescent bacteria are continuously illuminated, *A*. *katoptron* darken the light output by rotating the light organ downward, so that only the darkly pigmented back of the organ is exposed [31]. The luminous symbiont of *A*. *katoptron* is shown to be the bacterium, *Candidatus Photodesmus katoptron* (Gammaproteobacteria: Vibrionaceae) [32] and has genomic features in common with unrelated obligately dependent symbionts, such as insect endosymbionts [33].

Using low-light camera technology, we investigated nighttime behavioral patterns of bioluminescent fishes, in their natural environment, using minimal artificial light sources. These observations were made with no artificial lighting, since avoidance behavior from artificial illumination was readily observed. This study recorded nighttime schooling behavior of thousands of flashlight fish departing their shallow daytime resting caves and descending down the reef at night. Our results demonstrate that flashlight fish use their bioluminescent flashing to facilitate schooling at night. Flashlight fish schooling is unique in that some members of the school do not flash (potentially decreasing chance of predation), while still participating in group behavior. To further explore this phenomenon, we created a model of flashlight fish schooling. We show through the combined analysis of field video recordings and modeling that if a small cohort of fish become motivated to go in some direction (e.g. pursuing prey or evading a predator), the rest of the fish will follow, causing a rapid and coordinated change in the overall direction of the school.

## Results

### Fish observations

**Fig 2:**
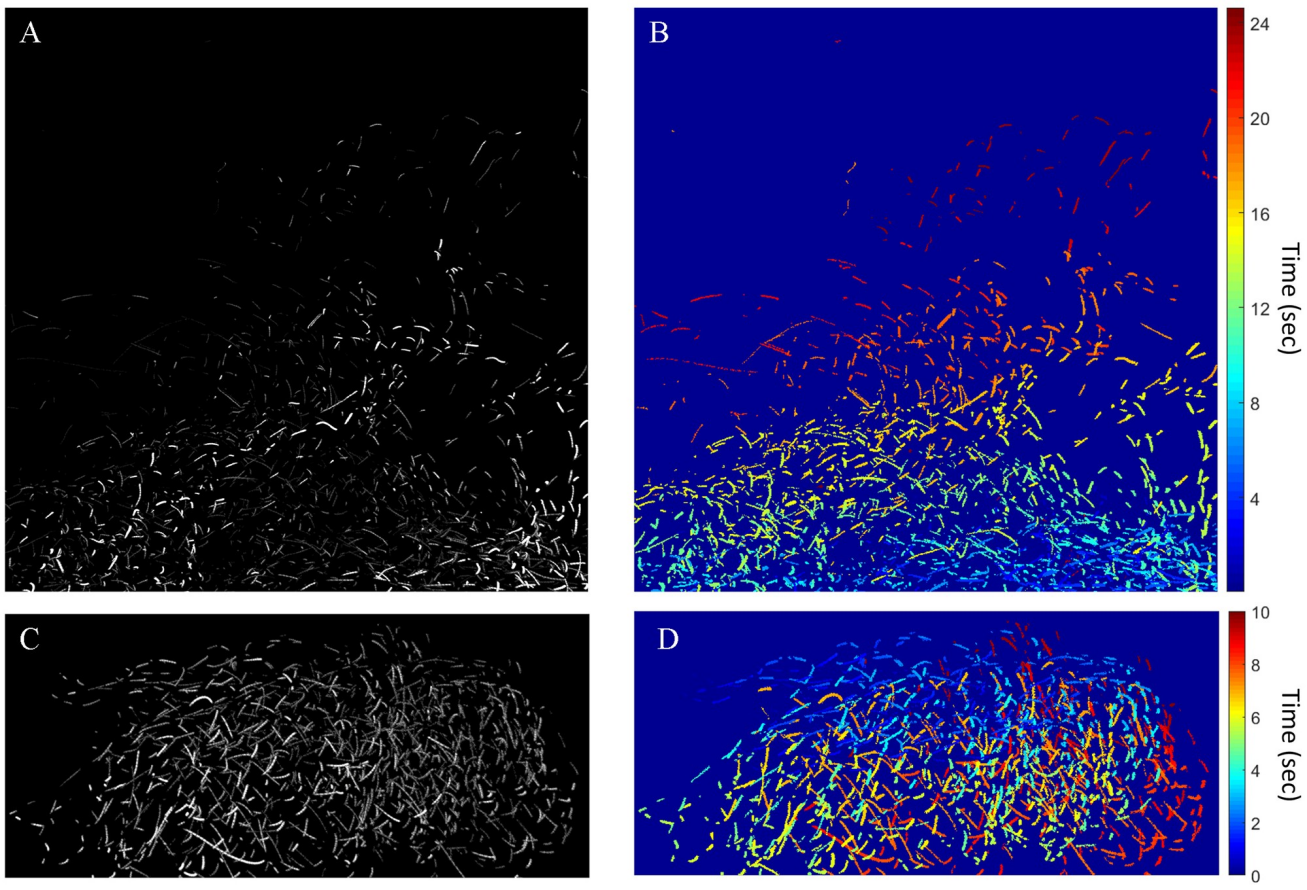
Flashlight fish recording. A) Time lapse image from the recorded video of flashlight fish. The different intensities are due to fish swimming at different depths. Closer fish are brighter than more distant fish. B) Time stamps, in seconds, corresponding to the flashes.

During two research expeditions in 2013 and 2016, large assemblages (hundreds to thousands of individuals) of the flashlight fish species *Anomalops katoptron* were observed and filmed off a remote tropical island in the Solomon Islands (S1 Fig). When diver-held lights were used to illuminate a school of flashlight fish, the fish quickly scattered to avoid this artificial light source (S1 Movie). In several recordings made without any artificial lighting, an entire school of *A*. *katoptron* was captured using a Hamamatsu Photonics ORCA-Flash4.0 V2 sCMOS camera (S2 Movie). Selected for analysis are two clips (25-second and 10 seconds) shot at 30 fps with a resolution of 2048x2048. Fig 2 shows a time-lapse of the two sets of recorded video with timestamps shown in seconds. From this dataset, localization of fish for every frame was conducted and tracking analysis was performed on each fish during the time it was flashing.

### School modeling

In order to model the movements of fish schools, we implemented an algorithm based on the interactions between simulated individuals in a 3-dimensional space, mediated through three forces: cohesion (the tendency to move towards nearby fish), alignment (the tendency to align to the direction of movement of nearby fish), and separation (the tendency to keep a certain distance away from other fish). Friction with the surrounding water was also modeled as a simple resistive force. These forces, taken together with relative weights based on real-world data obtained by previous researchers [34], and applied to a large number of simulated fish, produced complex schooling behaviors similar to those observed in the video data. To simulate the unique qualities of flashlight fish schools, the simulated fish were made to flash, so that they would be visible to others only while flashing. The parameters of this flashing, such as frequency and duty cycle, could be controlled by the user.

### Synchronous motion

**Fig 3:**
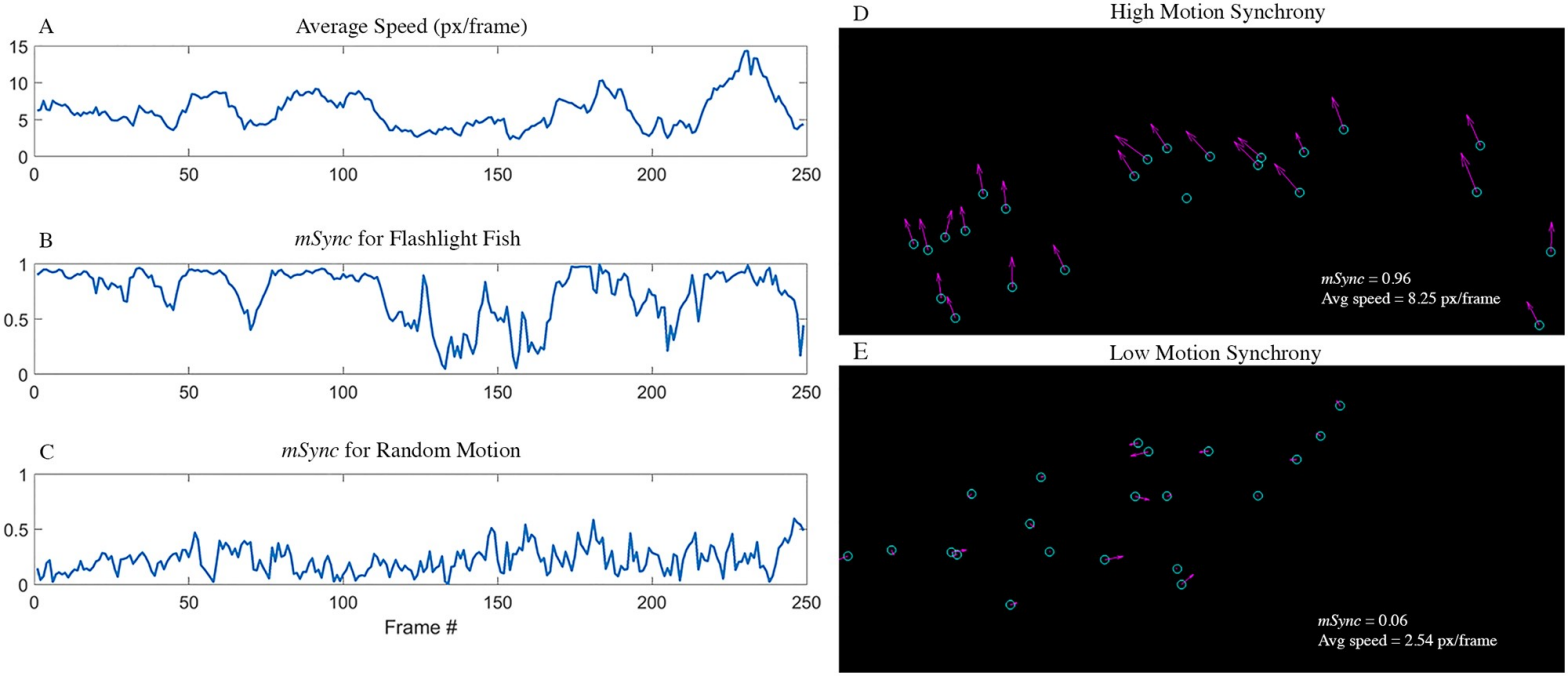
School motion synchrony. A) Average speed of all fish per frame. B) *mSync* computed per frame. We observe that when there is significant movement within the school, i.e. large average speed, there is motion synchrony. Low *mSync* values are observed when the school is almost at a standstill. C) *mSync* values if the fish were moving randomly. This plot was simulated with random fish movement and shows *mSync* is low for such a scenario. Contrasting this plot with B), we observe that the flashlight fish are moving with synchrony in direction. D) A frame from the video indicating high motion synchrony, corresponding to the red dashed line in plot (B). The blue circles indicate the flashing *Anomalops katoptron*, the purple arrows indicate the velocity of the fish. We can observe that there is high motion synchrony when there is significant movement within the school. E) A frame from the video indicating low motion synchrony, corresponding to the purple dashed line in plot (B). We can observe low *mSync* values are observed when the school is almost at a standstill.

**Fig 4:**
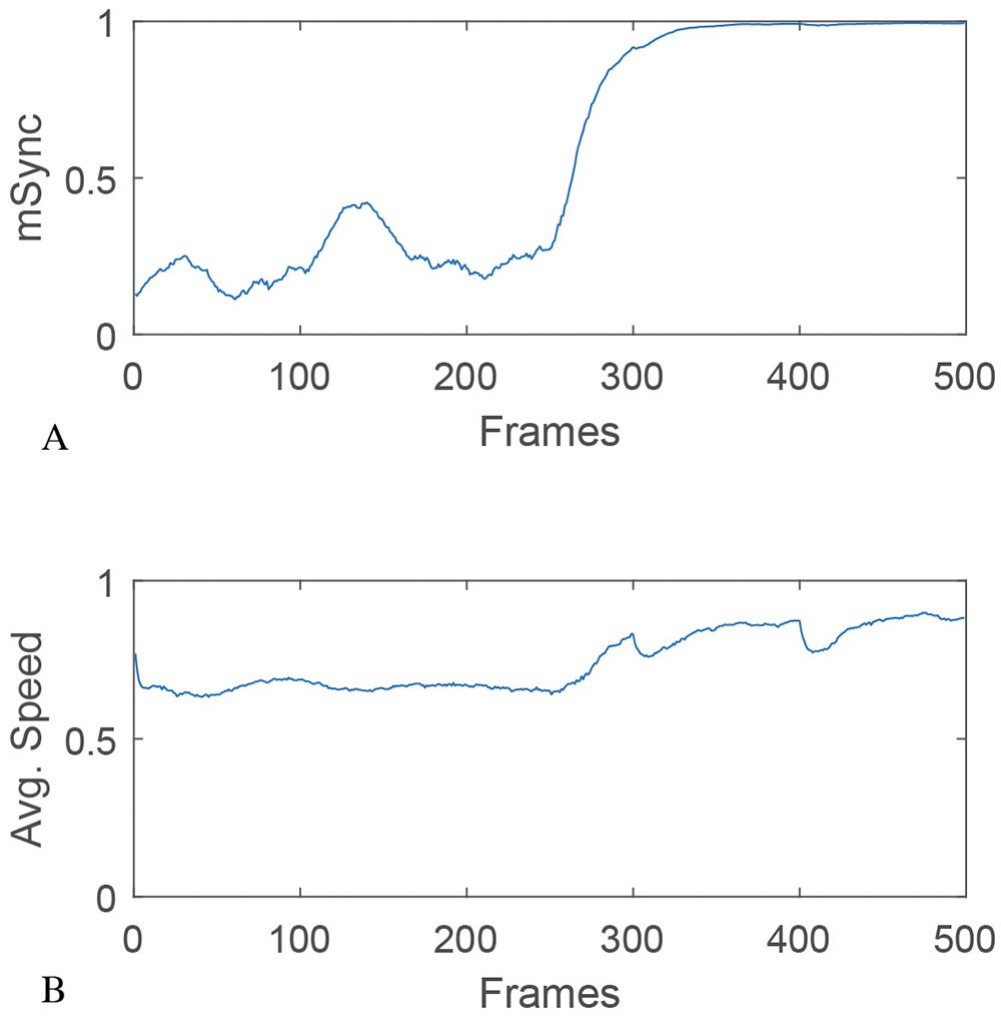
mSync and speed. Correlation between mSync and speed in the model data.

To test whether fish in both our model and in recorded video exhibit schooling behavior, we devised a metric, *mSync*, which measures the synchronicity of the fishes’ movements, and is based on the mean speed and velocity of all the fish in a frame. *mSync* is defined:

$$mSync(n)=\frac{\mu_{\overset{⃑}{v}(n)}}{\mu_{v(n)}}$$

where $\mu_{\overset{⃑}{v}(n)}$ is the mean velocity of all fish in frame *n*, and *μ*_*v*(*n*)_ is the mean speed of all fish in frame *n*. *mSync* is a value between 0 and 1, where 0 indicates shoaling and 1 indicates ideal schooling. Based on the video data (Fig 3, S2 Fig), we observe that when the fish are moving slowly (when the average velocity of the fish is low), they are shoaling, leading to low values of *mSync*. When the fish are moving faster, they switch to schooling, indicated by high values of *mSync*. The tendency of fish to school when moving quickly can also be observed in the school modeling data, to which *mSync* can also be applied (Fig 4, S3 Movie). To test the null hypothesis that the observed movement might have no synchrony, we simulate random fish movement and plot the corresponding mSync values (Fig 3C, S2C Fig). We compared the mSync values of the observed movement and the simulated random movement using the Wilcoxon Rank Sum Test [35] and obtained a p-value of the order of 10^−100^, rejecting the null hypothesis that the distribution of observed data and random movement are the same.

**Fig 5:**
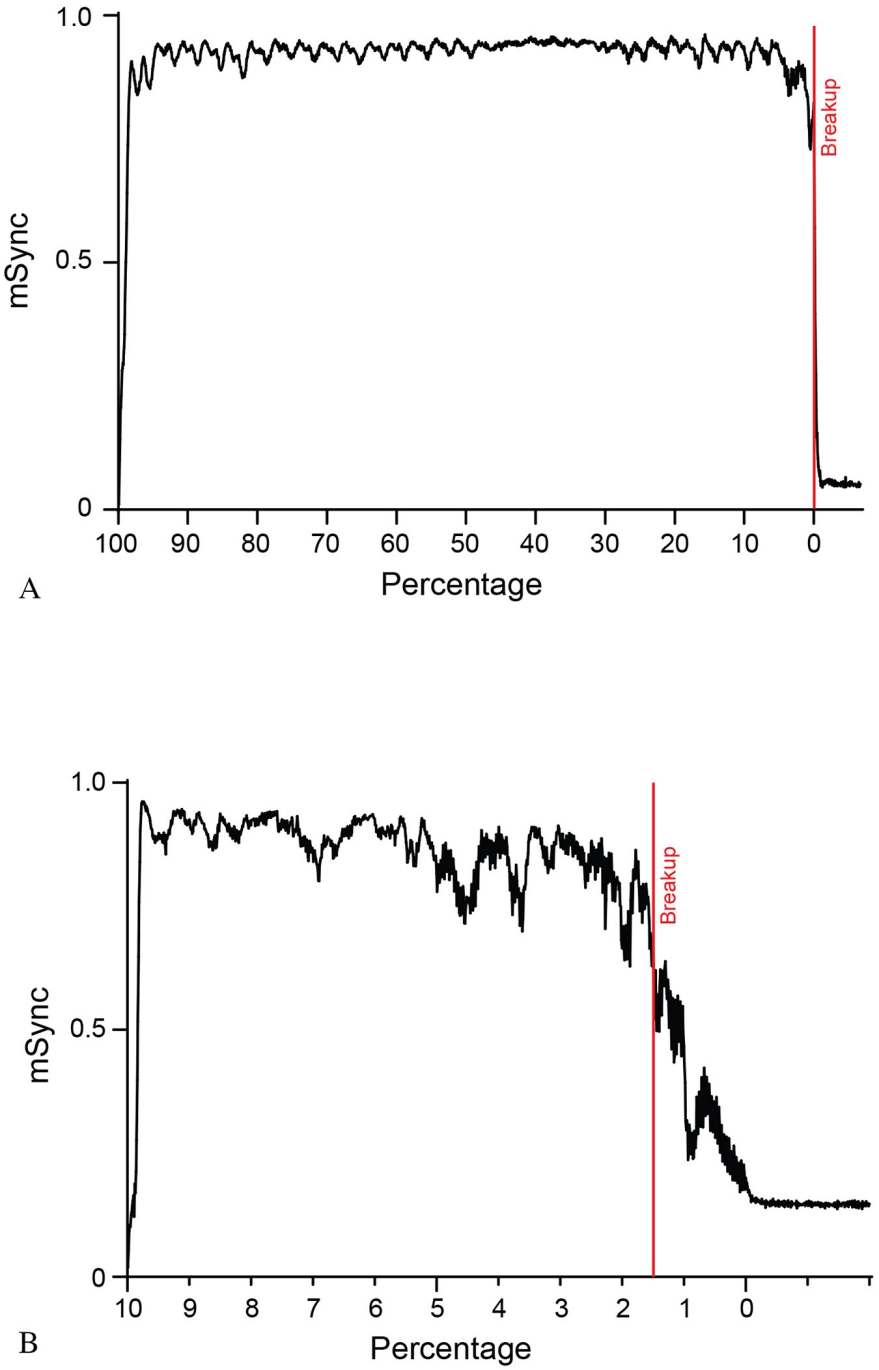
mSync vs. the proportion of flashing fish. A) Modeled by 5% intervals B) Modeled by 1% intervals.

Since flashlight fish can occlude their lights, presumably making themselves inconspicuous to conspecifics under low-light conditions and potentially disrupting or preventing schooling behavior, we used our algorithm to model the effects of these “dark fish” on schooling dynamics. As can be seen in Fig 5 and S4 Movie, *mSync* is relatively unaffected by the number of dark fish until they exceed 95% of the school–that is, less than 5% of the school needs to be flashing in order to maintain school structure.

As a first approximation, one might hypothesize that there is a strong selective advantage for fish to be invisible, because it reduces their likelihood of being eaten by predators. Since this is clearly not the case in our observations, there must be some other reason for fish to maintain their flashing beyond the level required for schooling. As it has been shown that flashlight fish use their bioluminescence for feeding [36], it seems likely that this is one reason they do not remain invisible. Another possible reason is predator avoidance through distraction. In video data of startled fish (S5 Movie), we observed the same “blink-and-run” behaviors seen in aquaria experiments on flashlight fish in the genus *Photoblepharon* [37]. Such evasive maneuvers are synchronized with flashing–when startled by light, the flashlight fish can be seen to flash their lights, then rapidly turn and dart away, then flash again, allowing them to misdirect potential predators. Further, the “swarming” luminescence we observed could be advantageous in that it might both serve to confuse a potential predator, as well as illuminate and reveal a potential predator to secondary predation, essentially functioning as a “burglar alarm” [38].

### Motivated fish

**Fig 6:**
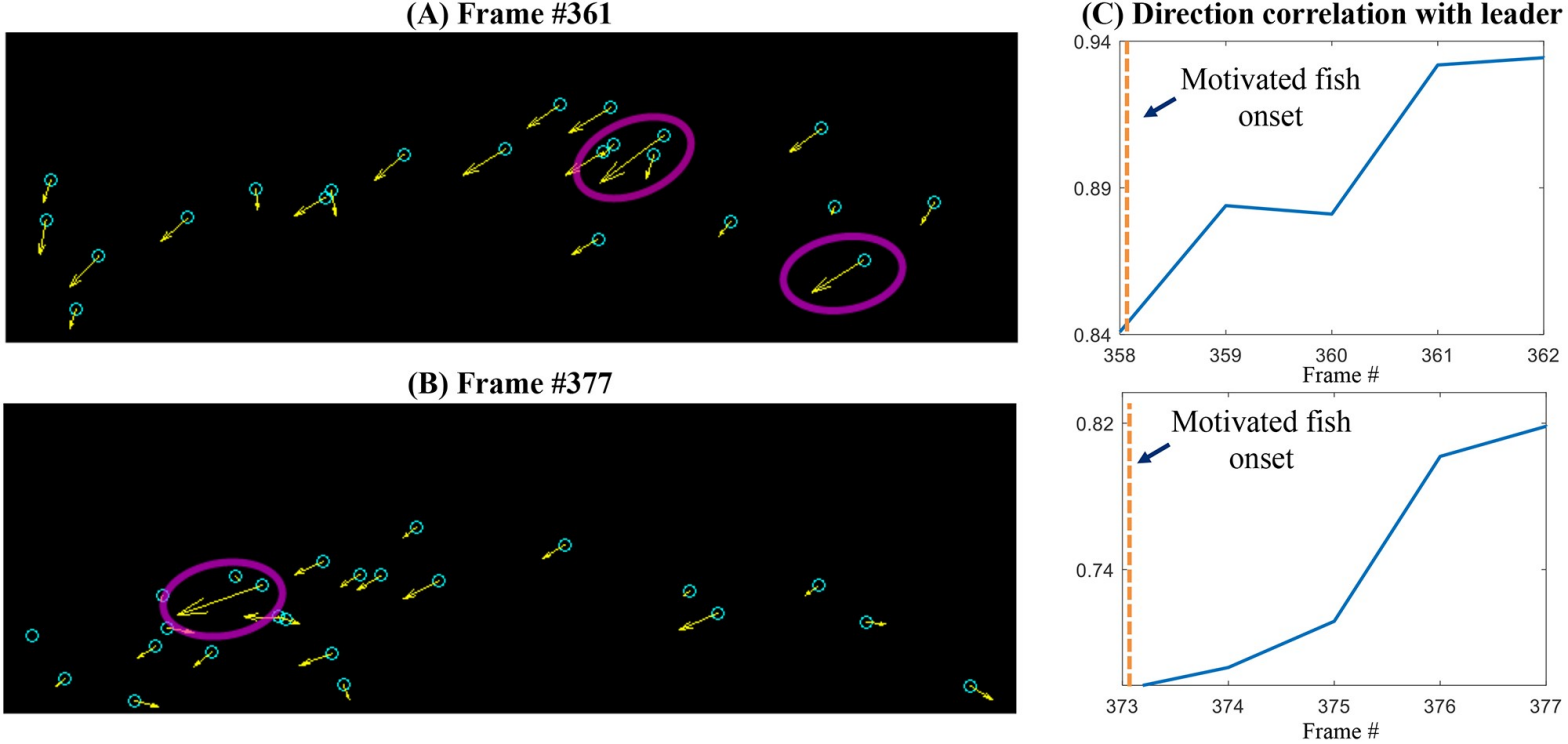
Motivated fish. (A,B) Frames from S2 Movie showing motivated fish who direct the school. The cyan circles indicate the flashing fish, the yellow arrows indicate the velocity of the fish. Longer arrows indicate higher speeds. The motivated fish are marked with purple ovals. The motivated fish move at higher speeds and the rest of the school align themselves to the direction of the leaders. (C) Plots showing correlation of direction of flashlight fish with the direction of the motivated fish. We can observe that after the onset of motivated fish, the correlation increases indicating that the rest of the fish are aligning with the motivated fish.

During the recorded swimming period, the school of flashlight fish changes direction multiple times. During these changes, we observed that 1–2 individual flashing fish would speed up along a particular direction and the rest of the school would soon align to their movement. We used our model to investigate this behavior and found that if only a few flashing individuals are motivated to move in a particular direction the rest of the school will soon follow, as shown in S6 Movie. Evidence of motivated fish can be observed in the real video data (Fig 6 and S7 Movie). To quantitatively represent the alignment of fish, we plot a directional correlation metric defined as follows:

$$\text{Direction}\;\text{correlation}\;\text{for}\;n^{th}\;\text{frame}=\frac{\sum_{i}\langle v_{L},v_{i}\rangle }{|v_{L}|\sum_{i}\left|v_{i}\right|},$$

where, 〈*,*〉 is the inner product between two vectors, *v*_*i*_ is the velocity of i^th^ fish in the n^th^ frame and *v*_*L*_ is the velocity of the motivated fish. When there are two motivated fish, *v*_*L*_ is considered as their average velocity. The value of direction correlation lies between 0 and 1. Higher values of correlation indicate the fish are aligned with the motivated fish. The direction correlation for a subset of frames in the S7 Movie is plotted in Fig 6C. We observe that the correlation increases after the onset of the motivated fish, indicating the alignment of the rest of the fish with the motivated fish. The flashing is crucial to convey the direction of motivated fish to the rest of the school.

### Flashing duty cycle

**Fig 7:**
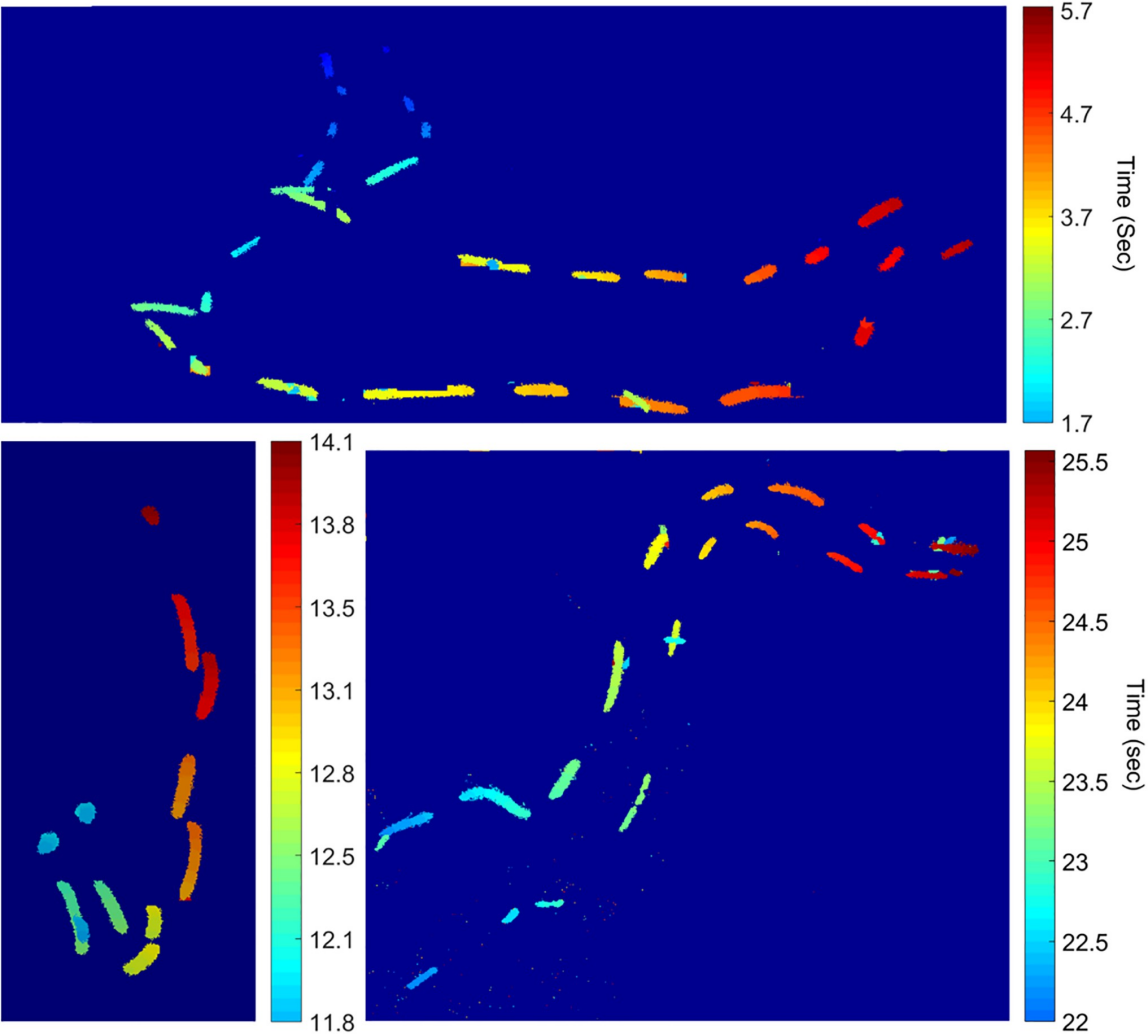
Synchronous swimming. Examples showing pairs of fish swimming synchronous to each other. The color bar shows progression of time in seconds.

**Fig 8:**
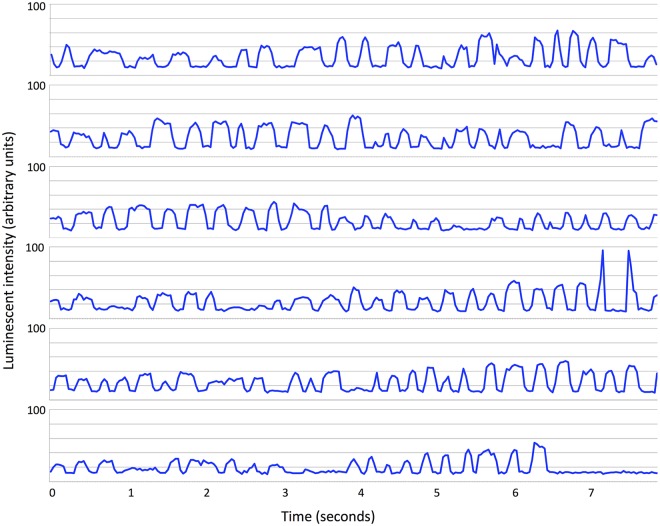
Six *Anomalops katoptron* tracked in the same time sequence as they bioluminesce within a school. Arbitrary units are maximum intensity values for each flashing fish derived directly from the raw 16-bit image data after background noise subtraction.

**Fig 9:**
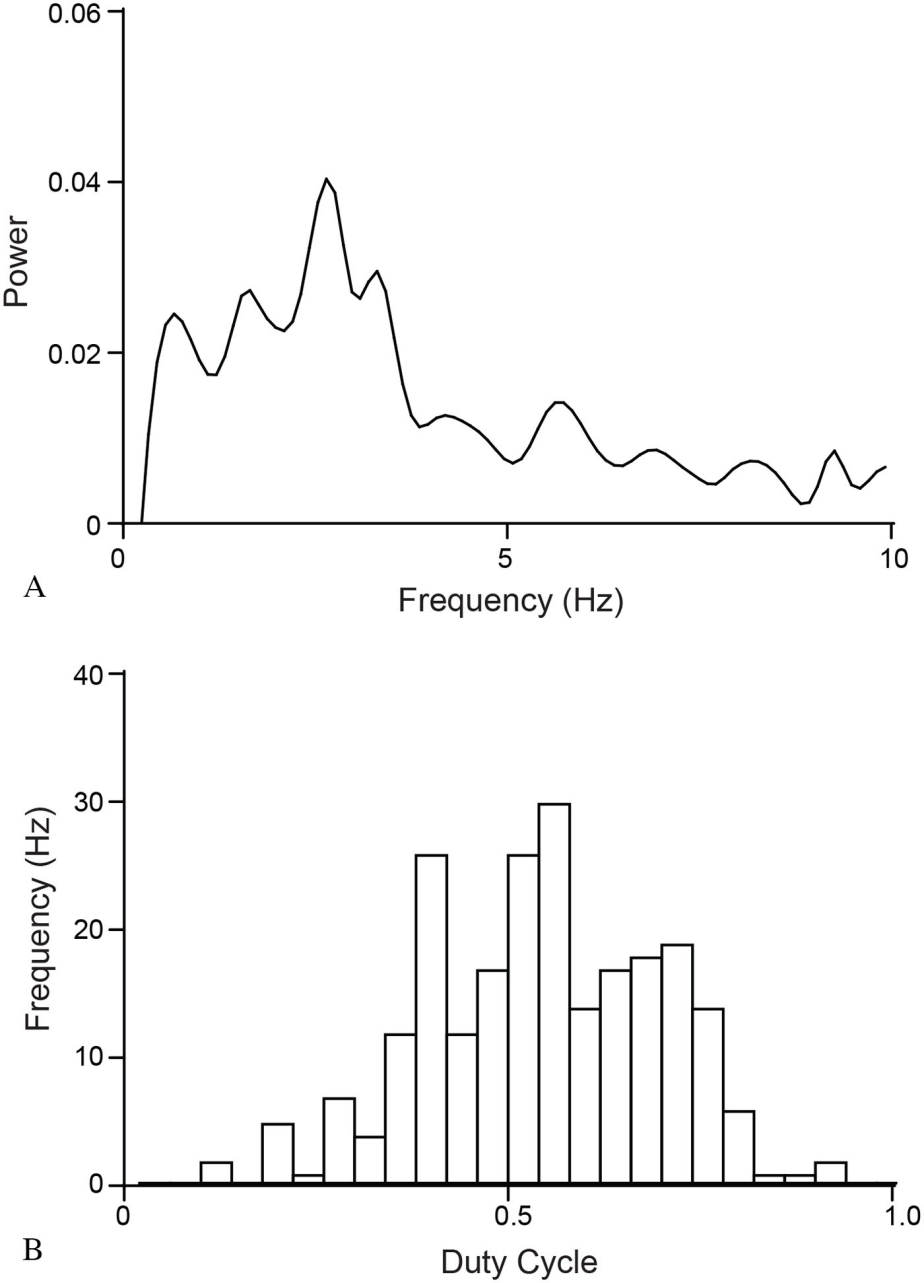
Analysis of characteristics of 234 flashes from 13 fish. A) Power spectrum of frequencies of flashes (averaged across all 13 fish), with a peak at approximately 3 Hz. B) Histogram of duty cycles of all 234 flashes, showing a center around 50%.

Analysis of the rate and duty cycle of the flashing in the video data can be seen in Figs 7 and 8. In the latter data, for example, the fish blinked at an average rate of 3.05 ± 0.3 Hz (the peak of the power spectrum in Fig 9A) on approximately-even intervals (shown by the histogram of duty cycles in Fig 9B), with average “on” and “off” times of 0.166s and 0.168s, respectively. Analysis in our model shows that schooling behavior (*mSync*) is insensitive to a wide range of these parameters. It seems likely therefore that these values are primarily related to feeding, and not to schooling.

#### Methods

Mborokua, Solomon Islands (9°1’12”S, 158°44’24”E), is an uninhabited, jungle-covered volcanic island located 30 km west of the Russell Islands (S1 Fig). It has dramatic vertical relief, from shallow bays to over 2000 m; due to its remote nature, it has experienced low levels of human impact. Filming via SCUBA took place on September 26–27, 2013 between 18:30–20:00 (approximately 1 week before a new moon) and on October 29, 2016 and November 2, 2016 between 19:30–21:00 (both sides of the new moon). On the evening of September 26, 2013, after shallow SCUBA filming, a Triton 3300/3 submersible was launched off the *R/V Alucia* (from 20:33–24:01) to observe the fish at greater depths.

**Fig 10:**
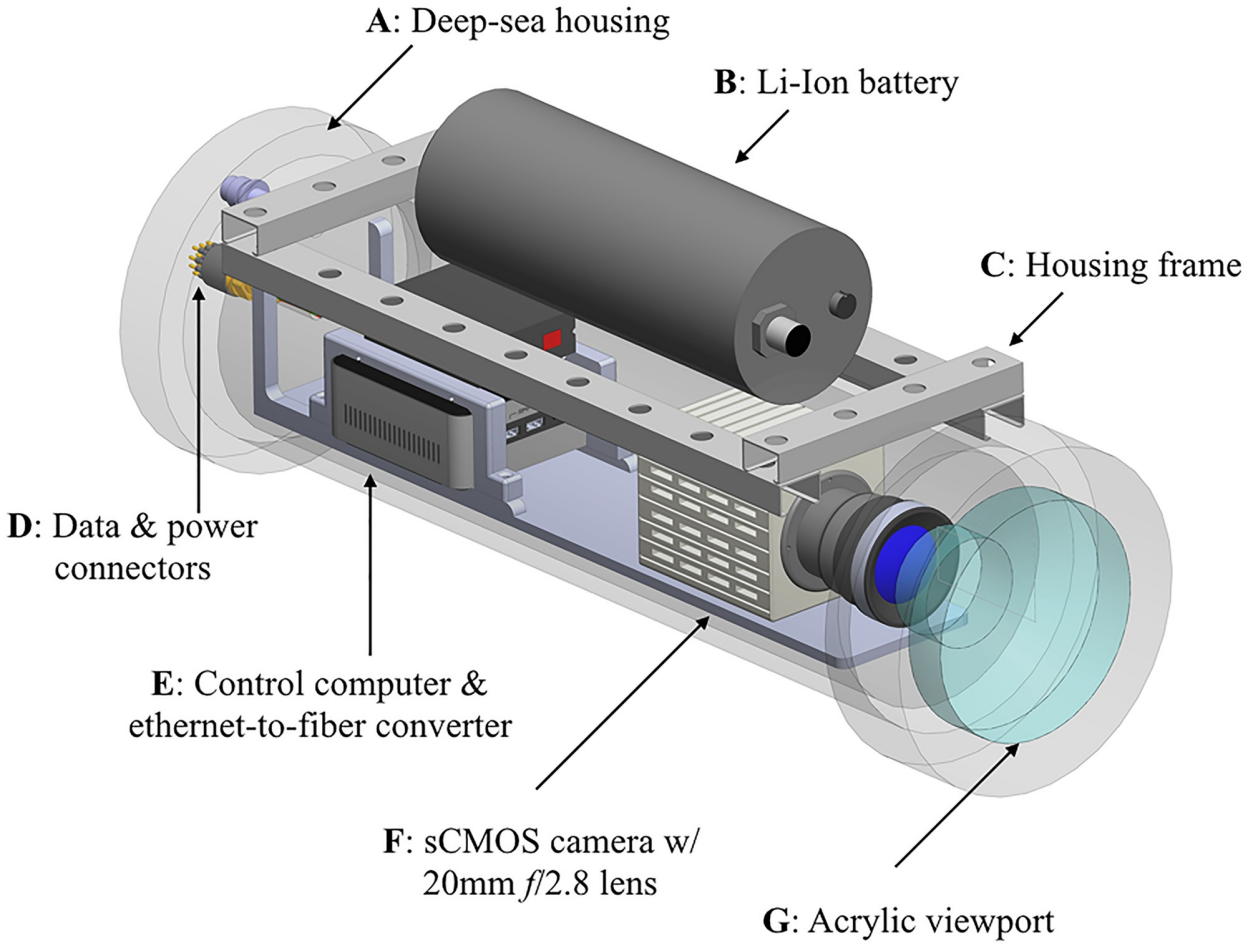
Three-dimensional model of the underwater low-light camera used for this study. This is based on an original design described in [39]. Components are A) housed inside a 22” long, 8” diameter 2500-meter rated housing that is B) powered by a separate 259 watt-hour lithium battery (SubC Imaging, Newfoundland CA). C) Housings are mounted onto a basic stainless steel frame along with floatation spheres to achieve neutral buoyancy (not shown). D) Communication and power is mediated through gigabit-Ethernet enabled fiber-optic and copper conductor connectors. E) An Intel NUC 5i5RYH computer supplied with a 4TB storage hard drive controls the F) Hamamatsu Photonics ORCA-Flash4.0 sCMOS camera outfitted with a 20mm *f*2.8 prime lens, which is G) optically coupled to the housing’s acrylic viewport.

Video imagery was collected using several types of underwater low-light imaging systems. All video used for data analysis was recorded with a Hamamatsu Photonics ORCA-Flash4.0 V2 sCMOS camera outfitted with a Nikkor f2.8 20mm prime lens and mounted in a custom underwater housing (Fig 10). Recordings recorded in S1 and S5 Movies were recorded with a Nikon D800 outfitted with a 50mm f1.4 Nikkor lens mounted in an Aquatica AD800 underwater housing.

### Image processing

With the Hamamatsu Photonics ORCA-Flash4.0 V2 sCMOS camera capturing at 30 fps, the emitted bioluminescence of the flashing fish uses a maximum of 1% of the camera’s 16-bit dynamic range. In such a low-light scenario, fixed-pattern noise (FPN) [40] of the sensor becomes a significant issue and needs to be corrected. FPN is caused by dark currents and refers to non-uniformity between pixels when the sensor is not exposed to light. FPN is an additive noise and, if estimated, can be subtracted out from each frame. Since the density of flashing fish is sparse, we can assume that each pixel in the sensor is exposed, most of the time, to a dark point in the scene. With this assumption, we estimated the FPN by taking an average of all the frames in the recorded video, and then subtracting this value from each video frame before further analysis was performed. The estimated FPN had mean of 100 (out of 16-bit or 65536 possible levels) and standard deviation of 5.

We are interested in the motion of the flashlight fish and, therefore, need to estimate the position of the fish in each frame. Each frame was first thresholded to form black and white (BW) images. The BW images were then passed through a morphological operation [41] to omit isolated noisy pixels. The centroid was calculated for remaining white pixel regions of a reasonable size (>20 pixels total area). These calculated centroids are the estimated positions of the fish in each frame and were used for tracking individual fish. The trajectory of each centroid was then tracked using an adaptation of MATLAB’s IDL Particle Tracking software [42]. The linking of positions between frames was done by selecting the most probable location within a given maximum radius. Only the fish that are flashing are tracked, as the positions of fish that are not flashing cannot be estimated.

### Specimen collection

Two specimens of *Anomalops katoptron* were collected via SCUBA in September 2013, using a hand net and Keldan underwater video lights, and are deposited in the AMNH Ichthyology Collection (AMNH 264834 n = 2) (http://sci-web-001.amnh.org/db/emuwebamnh/Display.php?i=4). Research, collecting and export permits were obtained from the Ministry of Fisheries and Ministry of Environment, Honiara, Solomon Islands. Specimens were collected with a hand net, transferred to the surface in plastic bags with seawater, and anesthetized/euthanized by exposure to the sedative MS-222 following approved protocols. This study was carried out in strict accordance with the recommendations in the Guidelines for the Use of Fishes in Research of the American Fisheries Society (https://fisheries.org/docs/wp/Guidelines-for-Use-of-Fishes.pdf) and approved by the American Museum of Natural History’s Institutional Animal Care and Use Committee (IACUC).

### Schooling simulation

The simulation of fish movement was performed with C++ code running on the CUDA platform to allow for massively-parallel processing and optimized floating-point mathematics. The simulated environment was an empty, infinite Hilbert space with dimensions X, Y, and Z. Positions and other 3-dimensional measurements within this space are expressed as 3-vectors 〈*X*, *Y*, *Z*〉. The representation of the fish consists of three such vectors: a position vector ***r***, a velocity vector ***v***, and an acceleration vector ***a***. Time within the stimulation proceeds in increments of Δ*t* seconds. At each frame, every fish is synchronously and simultaneously updated based on the state of the world in the previous frame. The movement of each fish is controlled primarily by three forces, referred to as Cohesion (***f***_*c*_), Alignment (***f***_*a*_), and Separation (***f***_*s*_). Each force is assigned a scalar weight which determines how strong it is in comparison with the other forces. The weights of these three forces (*w*_*c*_, *w*_*a*_, and *w*_*s*_, respectively) can be set by the user either manually or automatically at the beginning of the simulation, but are usually set to 7, 6, and 9 respectively, based on the weight and length of the flashlight fish [34]. In addition to these social forces, there are the internal forces of friction (***f***_*f*_) and speed control (***f***_*v*_) with their own weights (*w*_*f*_ = 1.05 and *w*_*v*_ = 5). Once each force is determined for a particular frame using its weight, the forces are summed. This net force is then applied to the fish, accelerating it such that $a=\frac{f_{tot}}{m}$, where *m* is the mass of the fish. Since the unit of mass in this simulation can be arbitrarily set to be the mass of a flashlight fish, this equation simplifies to ***a*** = ***f***_*tot*_.

In order to model the flashing of flashlight fish, a uniform random number in [0, 1] is selected for each fish on each frame, and if this number is less than a user-defined parameter *P*, the fish is considered flashing (and therefore visible) on that frame. In nature, *P* = 0.5, as the duty-cycle is 50%, but this can be set to different numbers to test different duty-cycles. There is also a parameter *D*, which is used in a similar fashion to determine whether a given fish will flash at all (whether or not it is “dark”). To do this, each fish is assigned a uniform random number *R* ∈ [0, 1] at the start of the simulation, and if *R* < *D*, the fish is “dark”, meaning that it will not flash under any circumstances. In nature, we assume *D* = 0.0, as all fish presumably flash. *D* and *P* can be made to change throughout the course of the simulation, such as in Fig 4, where *D* was changed.

The force of cohesion, ***f***_*c*_, is calculated with the expression $f_{c}=(\frac{s_{c}}{\left|s_{c}\right|}∙v_{max})-v$, $s_{c}=\frac{\sum_{k=1}^{n_{v}}r_{k}}{n_{v}}$ where ***r***_*k*_ is the position vector of the *k*^th^ visible fish, *n*_*v*_ is the number of fish that are not the current fish and that are visible for cohesion, and *v*_*max*_ is the maximum velocity a fish is allowed to have. The force of alignment, ***f***_*a*_, is calculated with the expression $f_{a}=(\frac{s_{a}}{\left|s_{a}\right|}∙v_{max})-v$, $s_{a}=\frac{\sum_{k=1}^{n_{v}}v_{k}}{n_{v}}$, where ***v***_*k*_ is the velocity vector of the *k*^th^ visible fish. The force of separation, ***f***_*s*_, is calculated with the expression $f_{s}=(\frac{s_{s}}{\left|s_{s}\right|}∙v_{max})-v$, $s_{s}=\frac{\sum_{k=1}^{n_{v}}d_{k}}{n_{v}},d_{k}=\frac{r-r_{k}}{{\left|r-r_{k}\right|}^{2}}$.

Each social force has its own “region of vision”, derived from [34] which determines what regions can be “seen” for the purposes of that force. Since Cohesion is mostly mediated visually, its region extends the furthest, but does not include 90° behind the fish. Separation, being mediated both by vision and by the lateral line, includes all but 60° behind the fish, and extends a much shorter distance. Since Alignment is mediated almost solely by the lateral line system, its region of vision only consists of the regions to the sides of the fish, missing 60° in front and behind, and extending a distance intermediate between Separation and Cohesion. To initialize the simulation, each fish is generated such that each dimension of ***r*** is a uniformly-distributed random floating-point number in [−10, 10]), ***v*** = 〈0,0,0〉, ***a*** = 〈0,0,0〉. In effect, the fish are placed randomly at rest inside a cube centered on the world origin with sides 20 units in length.

## Discussion

Bioluminescence, the production and emission of light from a living organism, is a phenomenon known to occur in over 700 genera of metazoans across the tree of life, with ~80% of these genera being marine [43,44]. The functions of bioluminescence are diverse, exemplified by remarkable morphological specializations that range from anatomically complex species-specific luminescent structures to variation in the biochemistry of the bioluminescent systems. Bioluminescence serves many functions (such as offensive, defensive and mate attraction/recognition) for marine organisms, and it frequently serves multiple roles for a single organism [44]. The flashlight fish (*Anomalops katoptron*) filmed in this study belong to a unique group of bioluminescent fishes that are found in both shallow reef and deep water habitats in the tropical Pacific, Atlantic, and Indian oceans [32].

*Anomalops katoptron* has been recorded from as shallow as 2 m to 400 m depth [45], with individuals moving into deeper water to feed at night (pers. obs.). The family Anomalopidae comprises nine species arrayed within six different genera, *Anomalops*, *Kryptophanaron*, *Parmops*, *Phthanophaneron*, *Photoblepharon*, and *Protoblepharon*, all of which are equipped with a pair of oval subocular bioluminescent organs (Fig 1) that the fish controls via rotating or covering to emit and occlude symbiotic, bacterially-produced light [46]. *Anomalops katoptron* and *Photoblephlaron palpebratus* are the two species of flashlight fishes that have been studied relatively extensively. *Anomalops katoptron* was formally described in 1856 by Pieter Bleeker [47], a Dutch ichthyologist and herpetologist. *Photoblephlaron palpebratus* was described in 1781 by Peter Boddaert, a Dutch naturalist [48] who noted how local inhabitants used the luminescent organ as a fishing lure, as bioluminescence lasts up to 8.5 hours after being extracted from the fish.

*Anomalops katoptron* utilize bacterially-mediated bioluminescent illumination from their subocular light organs to detect planktonic prey and the blink frequency of their light organs is exogenously controlled by ambient light levels [36]. A study using four individuals of *P*. *palpebratus* noted differences in flashing frequency between daytime and nighttime hours, leading the authors to hypothesize that flashing is related to predator avoidance, intraspecific communication and feeding [37]. In that study, fish kept in complete darkness exhibited circadian rhythms of blinking, with increased blinking frequency during daytime hours (37 blinks/min with each blink lasting 800 ms), versus 2.9 blinks/min with each blink lasting 260 ms during the nighttime hours. A combined field and tank study [36] found that *A*. *katoptron* exhibited blink frequencies of approximately 90 blinks/minute at night with equal on and off times. However, in experimental tanks, open light organs (bioluminescent) time increased when the fish were feeding compared to when prey was absent and the blink frequency decreased to 20% compared to blink frequency in the absence of planktonic prey [36]. *Anomalops katoptron* with functional bioluminescent organs have also been observed to be capable of feeding on adult *Artemia* in total darkness, whereas individuals with non-functional light organs are unable to feed at all under these conditions [observed by Rosenblatt RH in 31]. The multiplicity of usages of bioluminescence led Morin et al. [37] to conclude that flashing function in *P*. *palpebratus* is extensive and varied; including offensive, defensive and communicative capacities. Our results corroborate this body of information and further demonstrate the utilization of bioluminescent flashing to enable schooling in flashlight fishes under conditions of low to no ambient light.

While astral sources could play a role in schooling, we noted *A*. *katoptron* schooling behavior on overcast nights down to ~100m and *A*. *katoptron* has been reported to ~300-400m [45, 49]. To estimate astral at depth, we calculate the transmittance at 20, 30, and 100 m conservatively using a surface starlight value of 0.0002 lux (1.46 x 10^−10^ W/cm^2^) [50], and an absorption coefficient of (*0*.*0562* m^-1^) for non-turbid ocean water at 550 nm [51]. The transmittances at these depths were 7.5%, 2.1%, and 0.15%, giving absolute intensity values of 1.10x10^-11^, 3.01x10^-12^, and 2.26x10^-13^ W/cm^2^. There is only one outlier account (based on an aquarium study from over 50 years ago) that reports Pacific jack mackerel (*Trachurus symmetricus*) schooling at such low light levels [19].

Fish schooling has previously been shown to be based on attraction, alignment and repulsion, the former being mediated mainly by vision, and the latter two being mediated by the lateral line system [9,12–14]. Using low-light video cameras, we were able to record the bioluminescent flashes of the species *A*. *katoptron* in the wild in darkness (Fig 2). Analysis of the field video data, using *mSync*, a measure of school movement synchrony, showed that *A*. *katoptron* are indeed schooling at these low ambient light levels using bioluminescent flashing (Figs 3 and 4 and S3 Movie). Vision is critical to schooling in fishes, and schooling has been shown to confer a lower risk of predation [2–5], provide greater access to trophic resources [6], lead to reduced metabolic cost of transport [8], and allow for improved mate choice [7]. Our results show that flashlight fishes use bioluminescent visual flashing cues to school at night, as opposed to other fishes that can utilize vision to school only under conditions of sufficient ambient light [15–19].

Our computer model further suggests that schooling remains stable even when only a few percent of the fish in the school flash their bioluminescent light organ (Fig 5 and S4 Movie). It is possible that many fish in a school could participate in schooling behavior without actively flashing. We observed in the field videos a potential predator-avoidance strategy that has been termed “blink-and-run” behavior [37], in which fish present a brief flash then rapidly change direction before flashing again, presumably to confuse predators (S5 Movie). We note that this is only speculative as no predators were observed during these displays, and that such displays may have an entirely different function. Additionally, we used observations from our field video data and applied them to the model to show that a few fish, motivated to move in a particular direction can facilitate a change in direction for the entire school (Fig 6). We also show that bioluminescent flashing in *A*. *katoptron* exhibits a relatively small range of duty cycles and frequencies, with no appreciable correlation between the two (Figs 7–9). This raises interesting questions as to the collective behavior of flashlight fish flashing and to the ecological and communicative tradeoffs of flashing rates and the duration of leaving their bioluminescent organ open to the underwater world.

Understanding the kinetics of flashlight fish utilization of bioluminescence to school in the dark may also have practical applications in the design of schooling “robotic fish” that can be used for both environmental monitoring applications as well as to study behavior of other bioluminescent fishes by potentially mimicking luminescent signaling behavior to elicit species-specific signaling responses [52]. This technique might be particularly useful for studying luminescent signaling behavior in groups, such as lanternfishes (Myctophiformes), where we have shown that lineages characterized by species-specific photophore patterns (i.e., Myctophidae) are diversifying at a more rapid rate than lineages that lack species-specific patterns and that are hypothesized to utilize bioluminescence solely as a means of ventral counterillumination (i.e., Neoscopelidae) [53].

Our results also suggest the possibility that fish schooling via bioluminescence might be prevalent in deep-sea, mesopelagic habitats, in addition to shallow waters. Recent studies have shown that bioluminescence has evolved many more times in marine fishes than previously hypothesized [43], and that species-specific bioluminescent signaling is correlated with increased diversification rates in both deep open-ocean mesopelagic habitats [53] and shallow waters [36,37,54]. The unique, species-specific luminescent signals produced by species-rich shallow water (e.g., Leiogathidae, ponyfishes) and deep-sea mesopelagic fish lineages (Stomiidae, dragonfishes; and Myctophidae, lanternfishes) could also be used to facilitate schooling in habitats with low ambient light levels to complete darkness.

## Supporting information

S1 Fig Mborokua Island. Study site in the Solomon Islands.
(TIF)

S2 Fig School motion synchrony for second (10 s) video set. A) Average speed of all fish per frame. B) mSync computed per frame. We observe that when there is significant movement within the school, i.e. large average speed, there is motion synchrony. Low mSync values are observed when the school is almost at a standstill. C) mSync values if the fish were moving randomly. This plot was simulated with random fish movement and shows mSync is low for such scenario. Contrasting this plot with B), we observe that the flashlight fish is moving with synchrony in direction. D) A frame from the video indicating high motion synchrony, corresponding to red dashed line in plot (B). The blue circles indicate the flashing fish, the purple arrows indicate the velocity of the fish. We can observe that there is high motion synchrony when there is significant movement within the school. E) A frame from the video indicating low motion synchrony, corresponding to purple dashed line in plot (B). We can observe low mSync values are observed when the school is almost at a standstill.
(TIF)

S1 Movie Photophobic flashlight fish response. Nikon D800 video of *Anomalops katoptron* from Mborokua, Solomon Islands displaying photophobic response of fishes to external illumination.
(MOV)

S2 Movie sCMOS video of flashlight fish. Example of raw Hamamatsu Photonics ORCA-Flash4.0 V2 sCMOS video (compressed) of *Anomalops katoptron* school from Mborokua, Solomon Islands.
(MOV)

S3 Movie Modeled “normal” schooling behavior. Each fish in this scenario has a 50% flashing percentage. Green represents a flashing (visible) fish, while red represents a non-flashing (invisible) fish.
(MP4)

S4 Movie “Flash and run” behavior. Startled flashlight fish exhibiting “flash and run” behavior in which they flash their lights, then turn and swim away, then flash again, likely in an attempt to confuse potential predators.
(MP4)

S5 Movie Modeled schooling behavior over time. The value of D decreases, reaching 0% (no flashing fish) at frame 500.
(MOV)

S6 Movie Modeled schooling behavior of “motivated fish”. White dots represent motivated fish.
(MP4)

S7 Movie Schooling behavior of *Anomalops katoptron* video that exhibits “motivated fish” behavior. The cyan circles indicate the flashing fish, the arrows indicate the velocity of the fish. Longer arrows indicate higher speeds. The arrows of the motivated fish are colored magenta. The motivated fish move at higher speeds and the rest of the school align themselves to the direction of the leaders. The arrows of the fish that are aligned with the leaders are colored in yellow. Arrows of rest of the fish are colored in cyan.
(MP4)
