# Supplementary figures and images for: Bioluminescent flashes drive nighttime schooling behavior and synchronized swimming dynamics in flashlight fish
Source: PLoS One. 2019 Aug 14;14(8):e0219852. doi: 10.1371/journal.pone.0219852 (PMC6693688; doi:10.1371/journal.pone.0219852)

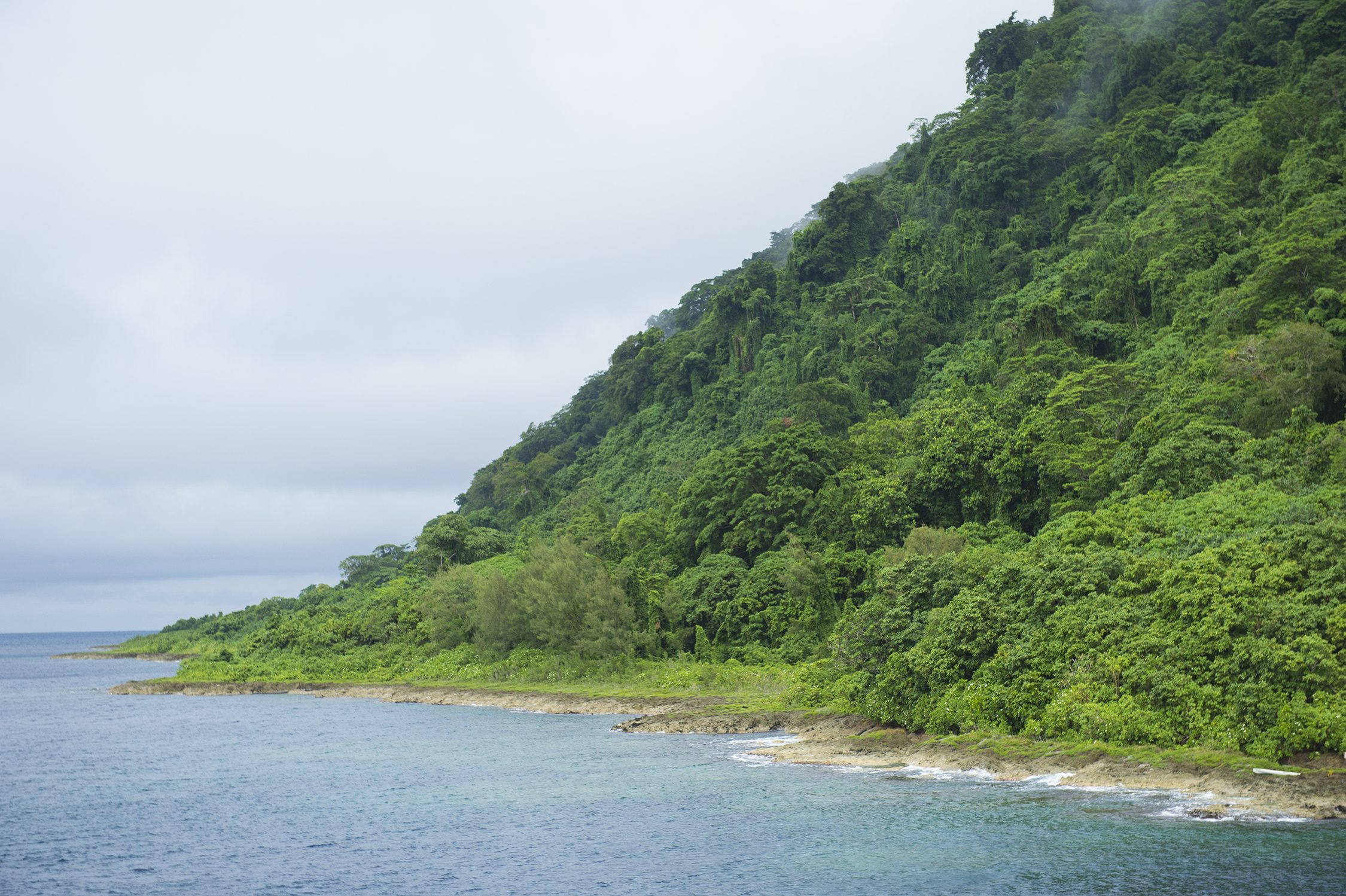

Supplement: S1 Fig — Study site in the Solomon Islands. (TIF) [file pone.0219852.s001.tif]

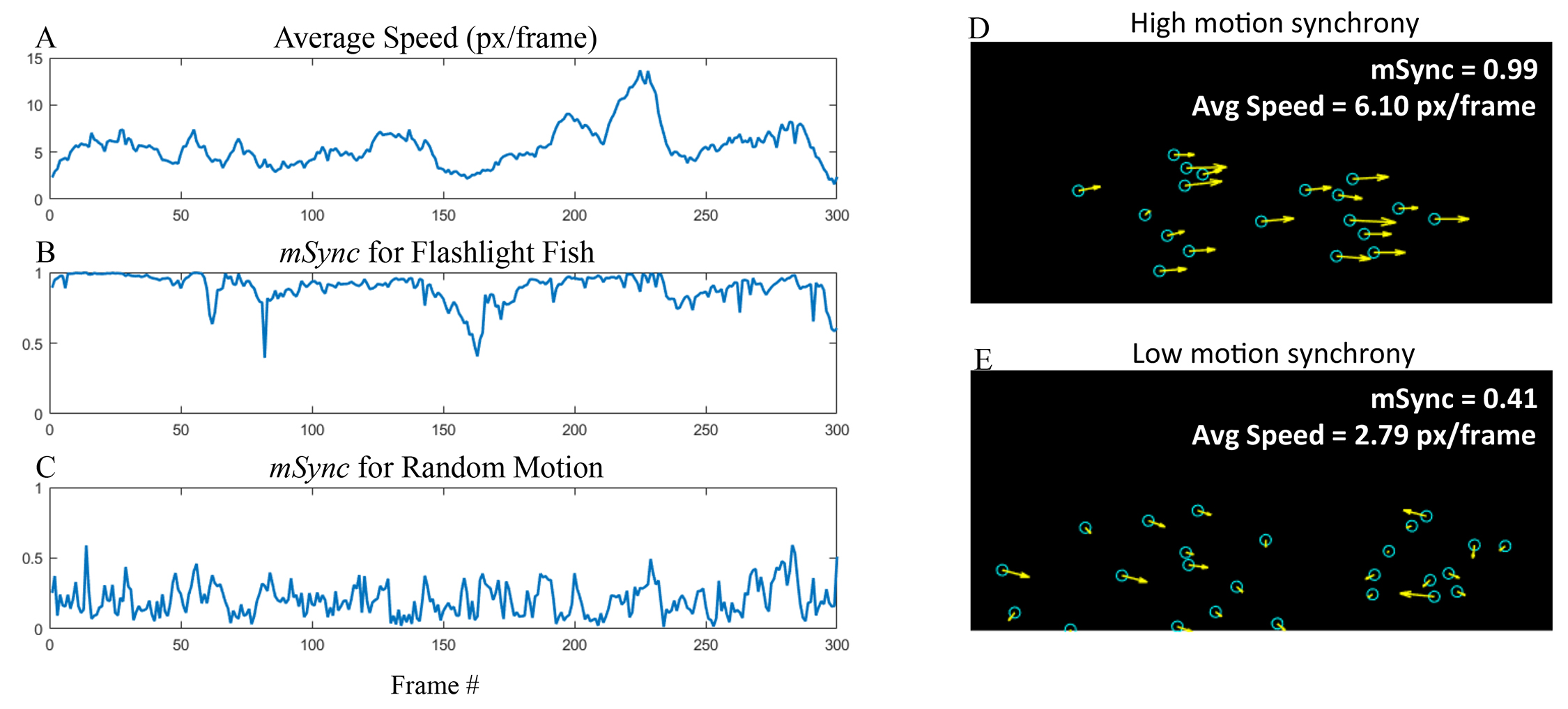

Supplement: S2 Fig — A) Average speed of all fish per frame. B) mSync computed per frame. We observe that when there is significant movement within the school, i.e. large average speed, there is motion synchrony. Low mSync values are observed when the school is almost at a standstill. C) mSync values if the fish were moving randomly. This plot was simulated with random fish movement and shows mSync is low for such scenario. Contrasting this plot with B), we observe that the flashlight fish is moving with synchrony in direction. D) A frame from the video indicating high motion synchrony, corresponding to red dashed line in plot (B). The blue circles indicate the flashing fish, the purple arrows indicate the velocity of the fish. We can observe that there is high motion synchrony when there is significant movement within the school. E) A frame from the video indicating low motion synchrony, corresponding to purple dashed line in plot (B). We can observe low mSync values are observed when the school is almost at a standstill. (TIF) [file pone.0219852.s002.tif]
